# Parenting and Temperament Influence on School Success in 9–13 Year Olds

**DOI:** 10.3389/fpsyg.2017.00543

**Published:** 2017-04-12

**Authors:** Purificación Checa, Alicia Abundis-Gutierrez

**Affiliations:** ^1^Department of Psychology, Faculty of Education Science, University of CádizPuerto Real-Cádiz, Spain; ^2^Department of Behavioral Sciences, Campus Los Valles, University of GuadalajaraGuadalajara, Mexico

**Keywords:** parenting styles, parent-children interactions, self-control, temperament, school success, academic results, school adjustment

## Abstract

Children spend a lot of time with their parents who are the first agents that educate them. The parenting style implemented in the family influences other contexts outside home such as the school. There is evidence that a positive parenting style has an influence on school success. However, there are other variables related to school success, for example, temperament. The influence of parenting decreases with age as children develop abilities to self-regulate without parents' external control. The aim of the present study was to evaluate the contribution of parenting style and temperament in 9–13 years old children on both academic performance and school adjustment skills. Our hypothesis was that not only parenting style is crucial to academic performance and school adjustment, but also temperament plays an important role in them. We used a Parenting Guide line questionnaire to evaluate parenting style, Early Adolescence Temperament Questionnaire-R to evaluate temperament; Health Resources Inventory to assess children's school adjustment, and academic grades, as indicator of academic performance. We were interested in testing whether or not the effect of parenting style on academic performance and school adjustment was mediated by temperament. We found that emotional and behavioral regulation mediates the relation between parenting and academic performance. These findings inform of the relevance of child's temperament on school success. Implications for education are discussed with emphasis on the importance of understanding students' temperament to promote school adjustment and good academic performance.

## Introduction

Parents, teachers, and the educational community, among other agents involved in the academic achievement of children and adolescents, are interested in knowing which variables affect school success. Broadly speaking, children's school success could be divided into academic performance (AP) and school adjustment. These two components are related to each other to some extent and both are required to cope with school demands. Much attention has been given to a range of variables that influence school success, for instance, individual differences in temperament (Blair and Razza, 2007; Valiente et al., 2007; Checa et al., 2008) and parenting style (Vitaro et al., 2006; Shute et al., 2011; RahimPour et al., 2015) have been reported to have an important impact in this matter.

One variable that has been linked to school success is parenting styles (PS). According to Darling and Steinberg (1993) PS is a compendium of attitudes toward the child during child rearing that create an emotional climate in which parents' behaviors are expressed. Some PS are based on physical punishment, lack of consistency and ineffective limit-setting while other PS use warmth, concern, consistency, positive discipline, and motivation (Bauermeister et al., 1995). It has been shown that school success is influenced by PS from infancy to late adulthood (Steinberg et al., 1989; Weiss and Schwarz, 1996; Zahedani et al., 2016). Furthermore, different patterns of PS have shown different associations with school success: PS based on warmth while maintain a structure and guidelines in the parent-child relationship is associated with academic achievement (Steinberg et al., 1989, 1992; Shute et al., 2011; Walker and MacPhee, 2011), whereas PS based on restrictive control or inconsistency is associated with low grades (Hillstrom, 2009; Parsasirat et al., 2013; Osorio and Gonzalez-Cámara, 2016). This link between PS and school success has been observed across various ethnic groups and socioeconomic backgrounds (Deslandes et al., 1997; Besharat et al., 2011; Ishak et al., 2012; Zahedani et al., 2016). Many studies have constantly shown that a positive PS is related to the development of an effective behavioral regulation during childhood. PS seems to be an environmental factor that influences childREN's development (Rinaldi and Howe, 2012). Children whose parents use discipline combined with warmth and dialogue, usually show more self-regulation (Weis et al., 2016) and higher self-esteem (Zakeri and Karimpour, 2011) than children raised by parents who exert a restrictive control. Additionally, it has been shown that PS based on warmth and discipline is related to less externalized problems in children (Eisenberg et al., 2005), while children that have negligent or excessively controlling parents are more likely to exhibit aggressive behavior and more externalized problems (Vitaro et al., 2006; Walker and MacPhee, 2011; Weis et al., 2016).

Other important variable that have been related to school success is temperament. Temperament is defined as individual differences in reactivity and regulation with a constitutional base (Rothbart and Bates, 2006). It has been proposed that the structure of temperament during childhood and adolescence is based on three broad dimensions: Surgency-Extraversion (SU), Negative Affect (NA), and Effortful Control (EC) (Rothbart and Bates, 2006; Rothbart, 2007). SU and NA describe individual differences in approach/avoidance reactivity, respectively, while EC defines individual differences in self-regulation, including inhibitory control and goal-oriented regulation of attention and activation (Rothbart and Derryberry, 1981). There is evidence in the literature of a consistent correlation between individual differences in some aspects of temperament and school success (Carey, 1998; Ellis et al., 2004; Blair and Razza, 2007; Valiente et al., 2007; Checa et al., 2008; Rueda et al., 2010; Checa and Rueda, 2011). Several studies highlight the importance of self-regulation or EC for academic achievement and school adjustment during childhood and pre-adolescence. Measures of the EC showed a positive correlation with academic outcomes, especially those related to reasoning (Coplan et al., 1999; Blair and Razza, 2007; Eisenberg et al., 2010; Valiente et al., 2013; Sánchez-Pérez et al., 2015) and skills involved in school adjustment (Checa et al., 2008; Rueda et al., 2010; Checa and Rueda, 2011). In contrast, NA has been associated with school adjustment problems. For example, aggressive behavior and anxiety, which are aspects of NA, are usually not compatible with social adjustment and school success. In a study conducted in Spain with 12-year old children, Checa et al. (2008) found that NA was associated with low AP and poor school adjustment skills, such as rule following, student-role understanding and tolerance to frustration. Likewise, some studies have shown that children exhibiting high levels of aggression (Clasen and Brown, 1985; Nelson et al., 1999; Schwartz et al., 2006) and children showing high levels of anxiety (Normandeau and Guay, 1998) showed poorer school success compared to their low NA peers. Regarding SU, the relation between SU and school success is not clear; while some studies show a positive relation between SU and school success (Farsides and Woodfield, 2003; Laidra et al., 2007), others have found no relation between these two variables (Checa et al., 2008; Deater-Deckard et al., 2009).

Most of the research in this field has studied the direct effect of either PS or temperament on school success without considering the possible relation or influence between them. The purpose of this study was, firstly to examine the relevance of PS and child's temperament to both AP and school adjustment, and secondly, to test the hypothesized role of temperament as mediator of the relation between PS and AP, and PS and different domains of school adjustment. Although, PS has been related to a broad variety of children's behaviors (Vitaro et al., 2006; Walker and MacPhee, 2011; Zakeri and Karimpour, 2011; Rinaldi and Howe, 2012), to our knowledge there is little investigation of the relation between PS and specific skills that promote school adjustment, such as rule following in the classroom, student-role understanding, socialization and tolerance to frustration; we analyzed these relations too. It was expected that a PS based on warmth and discipline was positively related to AP (Steinberg et al., 1989, 1992; Shute et al., 2011; Walker and MacPhee, 2011) and school adjustment skills. We also examined the consolidated relation between temperament and academic achievement (Coplan et al., 1999; Blair and Razza, 2007; Checa et al., 2008; Eisenberg et al., 2010; Rueda et al., 2010; Checa and Rueda, 2011; Valiente et al., 2013; Sánchez-Pérez et al., 2015). A positive correlation between AP and temperament factor of regulation (EC) was expected, as well as a negative correlation between school success and temperament factors of approach/avoidance reactivity (NA and SU). Children's temperament was examined as possible child-level mediators of the relation between PS and AP along with school adjustment, because temperament, in contrast to PS, is believed to have a constitutional base and could be less influenced by experience. We explored whether or not children's temperament (EC, NA and SU) mediates the relation between parenting practices and children's academic results and school adjustment. Specifically, we expected that EC, as aspect of self-regulation, and NA, as measure of regulation of negative emotions, mediated the relation between parenting and school success, as expression of an internalized regulation during early adolescence.

## Methods

### Participants

One hundred and eighty-nine children between 9 and 13 years of age participated in our study (49.73% male, *M* age = 10.26 years, *SD* = 1.25 years) along with their parents and teachers. Children had no diagnosis of neurological or clinical disorder. A total of 189 parents participated in this study. Participants came from families with similar socioeconomic status and were recruited from public elementary schools in Granada and Cádiz, Spain. The schools were part of a database of schools who participated in previous studies and expressed their willingness to participate in future research. The study protocol and recruitment procedures were approved by the Ethics Board of the University of Granada and University of Cádiz in accordance with the Spanish Ministry of Science and Innovation norms for research involving humans. Children's teachers provided information about the AP of the children participating in this study and parents provided information about PS and children's temperament. They were not paid for their collaboration.

### Measures

#### Parenting styles (PS)

Parents completed the Inventory of Parenting Guide line questionnaire [Inventario de pautas de crianza (IPC): (Bauermeister et al., 1995)]. The IPC consists of 37 questions aimed to measure parenting styles in daily common situations. Parents' responses are grouped into two factors: Coercive Parenting Style (CPS) and Sensitive Parenting Style (SPS). The former was obtained by 15 items that include the use of physical punishment, lack of consistency and ineffective limit-setting, while the latter was obtained by 22 items that include warmth, concern, consistency, positive discipline, and motivation toward their children. All items were responded on a 4-point Likert scale ranging from 0 (never or rarely) to 3 (very frequently). The internal reliability (measured by Cronbach's alpha) for each factor in our sample was: α = 0.74 for coercive CPS and α = 0.79 for sensitive SPS.

#### Temperament

Children temperament was evaluated using the parent-report format of The Early Adolescent Temperament Questionnaire-Revised (EATQ-R: Ellis and Rothbart, 2001) translated into Spanish by the Developmental Psychology Research group of the University of Murcia, Spain (GIPSE: Grupo de Investigación en Psicología Evolutiva. https://research.bowdoin.edu/rothbart-temperament-questionnaires/instrument-descriptions/the-early-adolescent-temperament-questionnaire/). The EATQ-R assess early adolescents (age 9–15) temperament and self-regulation via adaptation of scales used in studies with children and adults (Capaldi and Rothbart, 1992). The EATQ-R consist of 62 items referring activities and attitudes common to adolescents that parents had to rate by selecting the phrases which best described their children. All items were responded on a 5-point Likert scale ranging from 1 (almost always untrue) to 5 (almost always true). According to questionnaire scoring procedure, responses were grouped into three main factors: EC, SU, and NA. The internal reliability (measured by Cronbach's alpha) for the each factor in our sample was: α = 0.75 for EC, α = 0.38 for SU, and α = 0.72 for NA. We did not use SU temperamental characteristic in our analysis due to a low Cronbach's alpha. Future investigation has to improve the Cronbach's alpha of SU in order to explore the implication of this temperamental factor on school success.

#### Health resources inventory (HRI)

We used the Spanish version of the student self-report HRI (Juvonen et al., 1992) to assess children's competences related to their adjustment to school. The HRI consists in 31 items that evaluate children's skills in four domains: rule following (RF), student-role understanding (SRU), sociability (SO), and tolerance to frustration (TF). RF describes the student ability to function within the school environment constraints; SRU reflects children's understanding of the responsibilities and duties of a student as well as the behaviors associated with effective learning; SO refers to effective interpersonal skills; and TF measured the individual ability to cope with failure and other social pressures. All items were responded on a 5-point Likert scale ranging from 1 (never) to 5 (always). The internal reliability (measured by Cronbach's alpha) for each factor in our sample was: α = 0.77 for RF, α = 0.67 for SRU, α = 0.57 for SO, and α = 0.63 for TF.

#### Academic performance (AP)

Children's grades were the average of the scores in English, Language, and Mathematics. Since the information was gathered in June, AP was the result of the evaluation of the entire academic year (September to June). The grades were based in a 0–10 scale, where 10 is the maximum grade a child can obtain. Children's grades were provided by the school after parents' authorization and agreement to participate in the study.

### Procedure

Parents received an invitation to participate in the study via postal service. The informed consent letter, the IPC and EATQ-R questionnaires (instructions included) were also enclosed in the mail. Parents who decided to collaborate signed informed consent and completed questionnaires at home. Completion of the questionnaires took about 20 min and they were completed by one of the parents. Both, signed consent and completed questionnaires, were delivered by their children at the school in a sealed envelope (also provided). Parents were given a deadline of 20 days to deliver the information and documents required. In accordance to Declaration of Helsinki only parents that signed the informed consent participated in the study. Data of social competences related to school adjustment (HRI) were obtained from children in one ~20 min session at their school. Instructions to complete the HRI were given collectively and the questionnaires were completed individually by each participant in a pen-and-paper format.

### Analyses

Data were analyzed using SPSS Statistics software package version 17. We first tested correlation between variables to verify that our variables were suitable for mediation analysis. To assess whether or not the effect of different PS on AP and school adjustment skills was mediated by temperament, we used the macro created by Preacher and Hayes (2004). This macro was designed to test mediation effect and estimate the indirect effect of the independent variable on the dependent variable through the mediator by means of the Sobel test. Preacher and Hayes macro also provides a nonparametric bootstrap approach that randomly resamples the data to overcome the power problem introduced by asymmetries and other non-normality in the sample distribution (Preacher and Hayes, 2004).

## Results

The descriptive statistics on all measures are shown in the Table 1, except the temperamental factor of SU which was not considered in the analysis due to a low internal reliability. Further data analyses were performed using z-scores of all measures.

**Table 1:** **Descriptive statistics on all measures considered**.

**Measure**	**Minimum**	**Maximum**	**Mean**	***SD***
Age (years)	9.00	13.00	10.26	1.25
CPS (Coercive Parenting Style)	0.00	1.57	0.56	0.32
SPS (Sensitive Parenting Style)	1.00	2.73	1.89	0.36
EC (Effortful control)	2.13	4.86	3.31	0.52
NA (Negative affect)	1.17	4.08	2.50	0.58
RF (Rule following)	2.00	5.00	4.07	0.49
SRU (Student-role understanding)	1.83	5.00	4.03	0.64
SO (Sociability)	2.25	5.00	4.16	0.58
TF (Tolerance to frustration)	1.00	5.00	3.92	0.63
AP (Academic performance)	1.25	10.00	7.13	1.80

*N = 189 for all variables*.

Correlation results (Table 2) indicate that temperament, specifically EC and NA, is related to both PSs and school adjustment skills. There was a positive relation between EC and SPS, RF, SRU, and TF, indicating that children with high EC had less coercive and more sensitive parents, achieved higher grades in school and perceived themselves to be competent in RF, SRU, and TF. However, an inverse relation was found for NA, reflecting that children with high NA had more coercive and less sensitive parents, obtained lower grades and perceived themselves to be less competent in RF, SRU, and SO.

**Table 2:** **Statistically significant correlations between variables**.

	**Age**	**CPS**	**SPS**	**EC**	**NA**	**AP**	**RF**	**SRU**	**SO**
CPS	0.22								
SPS	−	−							
EC	−	−0.28	0.25						
NA	−	0.31	−0.16	−0.36					
AP	−0.41	−0.16	−	0.43	−0.22				
RF	−	−0.19	0.16	0.34	−0.16	0.41			
SRU	−	−0.16	0.15	0.36	−0.18	0.45	0.72		
SO	−	−	−	−	−0.15	−	0.53	0.52	
TF	−	−	−	0.20	−	0.34	0.67	0.67	0.36

*Significance level: p < 0.05*.

We were interested in testing whether or not the effect of different PS on AP and school adjustment skills was mediated by temperament. For this purpose 10 mediation models were analyzed (Table 8). Based on correlation results TF and SO were excluded for mediation analysis. In all models CPS or SPS were entered as predictor and AP, RF, and SRU as single dependent variables. 5000 bootstrap re-samples were used in all models. For CPS (Tables 3–5) we observed that the total effect of CPS on AP (*b* = −0.16, *p* = 0.027), RF (*b* = −0.18, *p* = 0.010) and SRU (*b* = −0.16, *p* = 0.026) were non-significant when EC were included in the model as mediator (AP: *b* = −0.04, *p* = 0.515, RF: *b* = −0.10, *p* = 0.166, SRU: *b* = −0.06, *p* = 0.360). Same pattern of results were observed on AP and SRU when NA was entered as mediator (AP: *b* = −0.10, *p* = 0.170, SRU: *b* = −0.12, *p* = 0.126), but not for RF: CPS kept significant after taking NA into account as mediator (*b* = −0.15, *p* = 0.046). The indirect effect of CPS through EC was statistically significant on AP, RF and SRU (Table 8). However, the indirect effect of CPS through NA was significant on AP but not on SRU neither on RF (Table 8). Regarding SPS as independent variable (Tables 6, 7), results showed that the total effect of SPS on RF (*b* = 0.16, *p* = 0.030) and SRU (*b* = 0.15, *p* = 0.044) was non-significant when EC (RF: *b* = 0.08, *p* = 0.262, SRU: *b* = 0.06, *p* = 0.386) and NA (RF: *b* = 0.13, *p* = 0.063, SRU: *b* = −0.12, *p* = 0.098) were included in the models as mediators. Nevertheless, mediation effect in SPS models, measured as indirect effect, was significant only on EC mediation analyses, but not on NA models (Table 8). Since both EC and NA were mediators of the effect of CPS on AP, we examined one more model including both temperament factors as mediators. Results showed (Table 9) that only EC is a mediator of CPS on AP, and NA effect disappeared.

**Table 3:** **Direct and total effects results for the effect of CPS on AP controlled for EC and NA**.

**Direct and total effects X = CPS, Y = AP**								
	**M = EC**				**M = NA**			
	***b***	**S.E**.	***t***	***p***	***b***	**S.E**.	***t***	***p***
Y/X	−0.160	0.072	−2.233	0.027	−0.160	0.072	−2.233	0.027
M/X	−0.208	0.052	−3.994	0.000	0.267	0.060	4.489	0.000
Y/M.X	0.555	0.092	60.004	0.000	−0.216	0.087	−2.482	0.014
Y/X.M	−00.045	0.069	−0.652	0.515	−0.103	0.075	−1.376	0.170

*Y/X, the total effect of X on Y; M/X, the effect of X on M; Y/M.X, the effect of M on Y controlling for X; Y/X.M, the direct effect of X on Y controlling for M*.

**Table 4:** **Direct and total effects results for the effect of CPS on RF controlled for EC and NA**.

**Direct and total effects X = CPS, Y = RF**								
	**M = EC**				**M = NA**			
	***b***	**S.E**.	***t***	***p***	***b***	**S.E**.	***t***	***p***
Y/X	−0.185	0.072	−2.585	0.010	−0.185	0.072	−2.585	0.010
M/X	−0.208	0.052	−3.994	0.000	0.267	0.060	4.489	0.000
Y/M.X	0.412	0.096	4.300	0.000	−0.130	0.088	−1.457	0.147
Y/X.M	−0.099	0.071	−1.389	0.166	−0.151	0.075	−2.009	0.046

*Y/X, the total effect of X on Y; M/X, the effect of X on M; Y/M.X, the effect of M on Y controlling for X; Y/X.M, the direct effect of X on Y controlling for M*.

**Table 5:** **Direct and total effects results for the effect of CPS on SRU controlled for EC and NA**.

**Direct and total effects X = CPS, Y = SRU**								
	**M = EC**				**M = NA**			
	***b***	**S.E**.	***t***	***p***	***b***	**S.E**.	***t***	***p***
Y/X	−0.162	0.072	−2.240	0.026	−0.162	0.072	−2.240	0.026
M/X	−0.208	0.052	−3.994	0.000	0.267	0.060	4.489	0.000
Y/M.X	0.464	0.096	4.841	0.000	−0.171	0.088	−1.939	0.054
Y/X.M	−0.065	0.071	−0.917	0.360	−0.116	0.075	−1.540	0.126

*Y/X, the total effect of X on Y; M/X, the effect of X on M; Y/M.X, the effect of M on Y controlling for X; Y/X.M, the direct effect of X on Y controlling for M*.

**Table 6:** **Direct and total effects results for the effect of SPS on RF controlled for EC and NA**.

**Direct and total effects X = SPS, Y = RF**								
	**M = EC**				**M = NA**			
	***b***	**S.E**.	***t***	***p***	***b***	**S.E**.	***t***	***p***
Y/X	0.160	0.072	2.190	0.030	0.160	0.072	2.190	0.030
M/X	0.184	0.053	3.485	0.001	−0.140	0.062	−2.249	0.026
Y/M.X	0.423	0.095	4.446	0.000	−0.160	0.084	−1.860	0.065
Y/X.M	0.080	0.071	1.124	0.262	0.135	0.072	1.870	0.063

*Y/X, the total effect of X on Y; M/X, the effect of X on M; Y/M.X, the effect of M on Y controlling for X; Y/X.M, the direct effect of X on Y controlling for M*.

**Table 7:** **Direct and total effects results for the effect of SPS on SRU controlled for EC and NA**.

**Direct and total effects X = SPS, Y = SRU**								
	**M = EC**				**M = NA**			
	***b***	**S.E**.	***t***	***p***	***b***	**S.E**.	***t***	***p***
Y/X	0.150	0.072	2.030	0.044	0.150	0.072	2.030	0.044
M/X	0.184	0.053	3.485	0.001	−0.140	0.062	−2.249	0.026
Y/M.X	0.468	0.095	4.932	0.000	−0.190	0.085	−2.246	0.026
Y/X.M	0.061	0.070	0.868	0.386	0.121	0.073	1.660	0.098

*Y/X, the total effect of X on Y; M/X, the effect of X on M; Y/M.X, the effect of M on Y controlling for X; Y/X.M, the direct effect of X on Y controlling for M*.

**Table 8:** **Indirect effects results for each model**.

			**Normal distribution results**						**Bootstrap results**			
**X**	**M**	**Y**	**Sobel value**	**S.E**.	**LL CI**	**UL CI**	**Z**	***p***	**Mean value**	**S.E**.	**LL CI**	**UL CI**
CPS	EC	AP^*^	−0.116	0.035	−0.185	−0.047	−3.30	0.001	−0.115	0.033	−0.187	−0.055
		RF^*^	−0.086	0.023	−0.144	−0.027	−2.88	0.004	−0.085	0.029	−0.147	−0.035
		SRU^*^	−0.097	0.032	−0.159	−0.034	−3.04	0.002	−0.096	0.029	−0.158	−0.046
	NA	AP^*^	−0.058	0.027	−0.111	−0.005	−2.13	0.033	−0.060	0.029	−0.123	−0.011
		RF	−0.034	0.025	−0.083	0.015	−1.36	0.175	−0.035	0.031	−0.099	0.023
		SRU	−0.046	0.026	−0.097	0.006	−1.74	0.081	−0.046	0.031	−0.114	0.008
SPS	EC	RF^*^	0.078	0.029	0.021	0.134	2.70	0.007	0.077	0.031	0.025	0.146
		SRU^*^	0.086	0.031	0.026	0.146	2.81	0.005	0.086	0.033	0.028	0.157
	NA	RF	0.022	0.016	−0.010	0.053	1.35	0.175	0.023	0.021	−0.005	0.074
		SRU	0.026	0.017	−0.008	0.061	1.52	0.129	0.028	0.021	−0.001	0.077

X, independent variable; M, mediator; Y, dependent variable; LL CI, lower level of 95% Confidence Interval; UL CI, upper level of 95% Confidence Interval.

* *Significance level: p < 0.05*.

**Table 9:** **Bootstrapping results of the indirect effect (IE) of CPS on Academic performance, through EC and NA as mediators in the same model**.

	**Academic performance *R*^2^ = 0.19, *F* = 14.23**			
	***b***	**SE**	***p***	**IE**
EC	0.53	0.10	0.000	−0.11
NA	−0.07	0.08	0.376	−0.02
CPS	−0.03	0.07	0.671	

## Discussion

Empirical studies provide useful guidelines for parents and educational practitioners to have a wider perspective of the variables that influence school success. In the present work we reveal relevant information about the link of parenting styles to school success through mediation of children's temperament. First, we expected that a Sensitive Parenting Style based on warmth and discipline was positively related to academic performance and school adjustment, whereas Coercive Parenting Style based on physical punishment, lack of consistency and ineffective limit-setting was negatively related to academic performance and school adjustment (Steinberg et al., 1989, 1992; Shute et al., 2011; Walker and MacPhee, 2011). Our data showed a correlation between SPS and school adjustment but we did not found relation between SPS and AP. However, as we expected, CPS was negatively related with both, AP and school adjustment. Secondly, we found a positive expected correlation between school success (AP and school adjustment) and temperament factor of regulation (EC) and a negative correlation between school success and temperament factors of reactivity (NA) (Carey, 1998; Ellis et al., 2004; Blair and Razza, 2007; Valiente et al., 2007; Checa et al., 2008; Rueda et al., 2010; Checa and Rueda, 2011). Finally, we expected that EC, as aspect of self-regulation, and NA, as measure of negative emotion regulation, mediated the relation between CPS/SPS and school success, because temperament, in contrast to parenting style, is believed to be less influenced by experience due to its constitutional base. We found that EC and NA mediate the relation between CPS and AP. However, when NA and EC are introduced as predictors of AP, only EC predicts AP. The relation between parenting (CPS and SPS) and school adjustment was also mediated by EC.

Parenting is one of the most important elements that affect child development and the influence of parenting on how children behave is not limited to the home environment (Vitaro et al., 2006; Walker and MacPhee, 2011). A PS centered on the use of physical punishment to control behavior, inconsistency in the established rules, and ineffective limit-setting, may possibly be related to the development of regulation skills based on fear and lack of confidence because parents did not provided a clear and efficient guidance on how to behave. In the other hand, it is likely that by combining warmth and positive discipline while parenting, parents teach their children some abilities that could be of use in school settings. For example, when parents exert some positive discipline at home, they teach their children some skills like rule following and tolerance to frustration that will help them in the process of adapting to school and learning academic content. Nonetheless, we did not find a positive association between SPS and academic results, rather we observed that CPS was negatively related to academic performance. These data suggest that the ability of parents to be loving and supportive, and yet maintaining an adequate level of discipline at home, is not the key to have better AP; conversely, the use of coercive strategies in parenting impacted negatively AP and school adjustment skills.

Our results also replicated a previously reported positive correlation between SPS and EC and a negative correlation between CPS and EC (Colman et al., 2006; Grusec and Davidov, 2007). It seems that CPS, through the use of punishment, inconsistency and not clear limits in the relation children-parent, failed in supporting the development of efficient self-regulation (EC) skills, while high degree of affection accompanied by expression of positive emotions, such as kissing and hugging (SPS), appeared to support and reinforce the development of self-regulation during childhood. Therefore, it is more likely that when parents are loving and caring for their kids, children will learn to control their behaviors and emotions in order to maintain positive parenting, and they are less likely to exhibit behaviors incompatible with such parenting style, like aggression, for instance (von Suchodoletz et al., 2011). Previous research have revealed a negative link between parental warmth and externalizing problems in children (Eisenberg et al., 2005; Jones et al., 2008). According to this, we also found a negative relation between the temperament factor of NA and SPS, and a positive correlation between NA and CPS. The impact of child's temperament on PS is not totally clear, because it is also possible that children's temperament plays a role on the type of parenting style parents display (Gault-Sherman, 2012; Kerr et al., 2012). However, we thought that an efficient self-regulation and management of NA could be learned during development through the parents' positive reactions and positive control toward the behaviors and emotions of their kids. Parents who dialogue with their children and encourage them to talk about their behaviors and emotions without punishment are likely to rear children who are relatively better to modulate their internal arousal and down-regulate themselves as required. The results discussed to this point indicate that the way parents educate their children at home was also reflected at school, where the children put their skills into practice. It seems that having coercive parents that exert physical punishment, lack of consistency and ineffective limit-setting, undermine the development of the appropriate abilities to success in school adjustment, and maintaining an adequate AP. Furthermore, a CPS appeared to be associated with both a deficient self-regulation and management of NA.

However, the data obtained from mediation analyses showed that self-regulation temperament factor EC, and NA mediate the relation between CPS and AP, and in the case of school adjustment skills, their relation with CPS and SPS were also mediated by EC. This set of results suggest that once EC and NA were taking into account children's AP and school adjustment skills were no longer explained by variations on PS, but by temperament, specifically EC and NA. There are studies that show a negative link between NA and problems with school adjustment (Normandeau and Guay, 1998; Nelson et al., 1999; Schwartz et al., 2006; Checa et al., 2008). We also found a negative association between individual differences in NA and both AP and school adjustment skills. Our data suggest that children with higher reactiveness to negative emotion have more difficulty following rules, understanding their role as student and socializing with peers. We also found that NA mediates the relation between PS and AP. Most of the children reproduce in the school the reacting pattern they exhibit at home. For example, negative reactions to life events could influence the adaptation to the classroom setting and AP, leading to increase discouragement and avoidance of sources of learning. The literature has shown the importance of the temperamental systems of self-regulation (EC) to school success across age (Carey, 1998; Ellis et al., 2004; Blair and Razza, 2007; Valiente et al., 2007; Rueda et al., 2010; Checa and Rueda, 2011; Sánchez-Pérez et al., 2015). Consistent with this we have found that the EC is positively related to both AP and school adjustment and also mediate the relation between parenting and school success. Moreover, our data show that when both EC and NA were introduced in the mediation analysis, only EC was a significant predictor of AP and school adjustment. As we mentioned earlier, the EC refers to the efficiency of child's self-regulatory abilities. Once children enter to school, the self-regulation abilities, more than parents monitoring, become a key aspect that support children in fulfilling with school demands. For example, when children are in the classroom, they have to use self-regulatory abilities without their parents control to regulate their level of activation when they start a new activity, or when they have to pay attention to their teacher, or when they must ignore distractors to concentrate in a specific task. In such situations, children with higher EC seem to be more efficient in the use of self-regulatory abilities, which in turn increase the opportunity for effective learning and the possibility of obtaining a good grade. Our results also showed that children with higher EC are more likely to have less trouble following rules, understanding their role as student, socializing with peers and tolerating frustration. Therefore, they may be experiencing less difficulty adapting to the classroom setting and thus more easily participating in the classroom routine as well as better approaching to potential sources of learning.

In sum, during the course of childhood, development of regulation of behavior and emotions occurs, first, with the assistance of an external source of regulation (usually exerted by parents), to eventually evolve to self-regulation (child's voluntary control) (Kopp, 1982). The influence of the external control seems to decrease as children grow up and improve their self-regulation abilities in the absence of their caregivers. According to our results, coercive parents, as external control agents, did not manage to successfully support their children to develop adequate strategies to regulate themselves in both neutral and emotional situations. Although self-regulation follows a long course of maturation, it is expected that at the age range of our sample children have developed self-regulation abilities that enable them to behave in an adequate manner without an external source of regulation. Hence, self-regulation abilities, more than parenting or NA, is what we observed to have a stronger influence on school success.

The effect of EC found in our study can have important implications in the education process. It is vital that both teachers and parents understand how individual differences in temperament, specifically EC, impact children's AP and school adjustment. Our results suggest that a PS had not a direct influence on the AP, neither on the competences children have to develop to cope with school adjustment. However, CPS diminish academic success. We believe that parents should increase their awareness of how their children's temperament relates to the way they react to academic context and schooling experience. Such understanding is likely to improve the way parents support their children to develop the appropriate abilities to face school demands. Similarly, schools should also ponder the key role of self-regulation on school success and the indirect influence of parenting on this important aspect of children's development. We consider relevant to emphasize the mediation role of self-regulation on school success in order to promote interventions in school-age children that target the regulation of behavior and emotion to improve academics results and enhance school adjustment.

Although our study indicate that self-regulation mediates the relation between parenting and school success, future research is needed to clarify if the influence of PS on school success goes from being direct to indirect during specific periods of self-regulation development. A cross-sectional and/or longitudinal study with a wider range of age could clarify this aspect. If a specific period during development is related to differences in how parenting and self-regulation skills influence school success, then more accurate and efficient interventions can be design in order to improve self-regulatory capacity on children (internal control) and/or promote positive parenting style (external control) that support school success.

## Author contributions

AA and PC design of the work and analysis, or interpretation of data for the work. AA and PC revised the work critically for important intellectual content; AA and PC approval of the version to be published; and AA and PC: the questions of the work are appropriately investigated and resolved. PC: Conception of the work and the acquisition of the data.

### Conflict of interest statement

The authors declare that the research was conducted in the absence of any commercial or financial relationships that could be construed as a potential conflict of interest.
